# Guarding Our Guardians: Navigating Adverse Reactions in Healthcare Workers Amid Personal Protective Equipment (PPE) Usage During COVID-19

**DOI:** 10.7759/cureus.58097

**Published:** 2024-04-12

**Authors:** Swetalina Pradhan, Nirav Nimavat, Nidhi Mangrola, Shruti Singh, Pallavi Lohani, Gowthamm Mandala, Rajesh Kumar, Nishi Sinha, Sunil Kumar Singh

**Affiliations:** 1 Dermatology, All India Institute of Medical Sciences Patna, Patna, IND; 2 Community Medicine, Gujarat Adani Institute of Medical Science, Bhuj, IND; 3 Community Medicine, Kiran Medical College, Surat, IND; 4 Pharmacology, All India Institute of Medical Sciences Patna, Patna, IND; 5 Community Medicine, Madhubani Medical College, Madhubani, IND; 6 Biological Health Sciences, Purude University, West Lafayette, USA

**Keywords:** prevention measures, healthcare worker safety, adverse allergic reaction, covid 19, personal protective equipment (ppe)

## Abstract

The widespread utilization of personal protective equipment (PPE) during the COVID-19 pandemic has been crucial for reducing transmission risk among healthcare workers (HCWs) and the public. However, the extensive use of PPE has brought about potential adverse reactions, particularly among HCWs. This study aims to investigate the prevalence and characteristics of adverse skin reactions associated with PPE use among different categories of HCWs, including faculty, residents, and nursing officers (NOs), in a dedicated tertiary care COVID-19 hospital.

The study design was a hospital-based cross-sectional analytical study conducted over one month, involving a total of 240 participants. The participants were required to complete a pre-tested semi-structured questionnaire that covered demographic information, PPE-related data, preventive measures, observed reactions, and self-management strategies.

Results indicated that adverse skin reactions were common among HCWs, with reactions reported by all participants. The most commonly used PPE included N95 masks, goggles, gloves, face shields, isolation gowns, and medical protective clothing. Excessive sweating (60% residents, 21.1% NOs, and 16.25% faculties), facial rash, dry palms (>70% of HCWs), and itching were among the most prevalent adverse reactions. Urticarial lesions (28.5% among NOs), pressure marks and pain (100% on the cheek among all HCWs), fungal infections (18.5% among residents at the web space of fingers), and skin breakdown were also reported. Factors such as age, gender, pre-existing skin problems, and oily/acne-prone skin history were found to be significantly associated with adverse skin reactions.

In conclusion, the findings highlight the common adverse reactions reported by HCWs during the use of different PPEs. Certain steps taken by HCWs for the prevention of adverse reactions due to PPE emphasize the importance of tailored preventive measures and strategies to mitigate these adverse reactions, such as proper PPE selection, well-fitting equipment, regular breaks, and appropriate skincare practices. These insights contribute to the development of guidelines for optimal PPE usage and support the well-being of HCWs in their essential roles.

## Introduction

The global impact of COVID-19 led to the widespread use of personal protective equipment (PPE), including masks and gloves, among healthcare workers (HCWs) and the public. PPE acted as a crucial barrier against the virus, reducing transmission risk. Adherence to PPE guidelines was stressed for HCWs, particularly those in direct contact with infected individuals. Understandably, high PPE use prevalence was observed in heavily affected regions [1-3].

While necessary, using PPE comes with potential adverse reactions. Common among HCWs are skin issues like dermatitis, itching, erythema, and acne. Prolonged PPE use, especially on the face and hands, can lead to pressure, friction, and discomfort [4]. Respiratory difficulties, especially with N95 masks, and heat stress in warm environments are widespread. The HCWs might also experience dizziness and nausea wearing Level 3 barrier protection equipment [5,6]. Other observations reported common adverse effects from PPE use, like burning, stinging, redness, moisture, raised skin lesions, and skin flaking [7].

To mitigate these adverse reactions, several preventive strategies have been identified, such as proper selection and fit of PPE, regular breaks and rotation, moisturization, hydration, and more. Swift identification and treatment of reactions is vital, with consistent monitoring and training crucial for optimal PPE usage and minimal adverse effects. However, research reveals limited adoption of preventive measures against PPE-related reactions. Encouraging HCWs to report discomfort and reactions is imperative. Early identification allows for timely interventions, including alternate PPE, preventive steps, and medical attention if needed [8-10].

Understanding and documenting these reactions is vital for PPE users' safety. The research raises awareness about risks, aids authorities in informed decision-making for PPE selection and usage, and contributes to guideline development. Identifying high-risk groups enables targeted interventions. Studying reactions aids future pandemic preparedness and tailors' recommendations, enhancing PPE effectiveness. Therefore, with this study, we intended to study adverse reactions in a healthcare center to provide evidence for HCWs to prevent and treat such issues during PPE use.

## Materials and methods

This hospital-based cross-sectional analytical study aimed to assess the prevalence and characteristics of adverse skin reactions associated with PPE use among HCWs in a dedicated tertiary care COVID-19 hospital. The study was conducted over one month, from July to August 2020, during the peak of the pandemic. The primary goal was to shed light on the extent of PPE-related adverse effects on HCWs and to identify key contributing factors.

Population and sampling

The study targeted a diverse group of HCWs from one institute (All India Institute of Medical Science, Patna), including faculty, residents, and nursing officers, who were actively involved in the care of COVID-19 patients within various sections of the hospital, such as the flu clinic and direct patient management areas. The sampling frame included 160 faculty members, 790 NOs, and 350 residents, totaling approximately 1,400 potential participants. The sample size was estimated based on previous studies, aiming for 178 participants to ensure a 95% confidence interval and 10% margin of error, factoring in an expected 50% response rate.

Questionnaire development and validation

A novel questionnaire was developed specifically for this study (Cronbach’s Alpha>0.7), drawing upon an extensive review of the existing literature on PPE usage and its potential adverse effects on healthcare professionals. The questionnaire underwent a rigorous validation process to ensure its relevance and comprehensiveness in capturing the necessary data. This process involved a panel of experts in the fields of dermatology, infectious diseases, and occupational health, who reviewed and provided feedback on the questionnaire's content.

Following validation, a pilot test was conducted among a select group of healthcare professionals not included in the main study. This pilot test aimed to assess the clarity, relevance, and overall effectiveness of the questionnaire in capturing the intended information. Feedback from the pilot test was incorporated into the final version of the questionnaire, ensuring its suitability for the study's objectives.

The finalized semi-structured questionnaire covered a broad range of topics, including demographic information, detailed PPE-related data, preventive measures adopted, observed reactions, and self-management strategies. Distribution was facilitated via Google Forms through WhatsApp and email to maximize reach and participation. A 15-day period was allocated for questionnaire submission, with reminders sent to encourage responses.

Ethical considerations

Ethical approval for this study was granted by the institutional review board (AIIMS/Pat/IEC/2020/518), with all procedures performed in accordance with the ethical standards of the committee and with the Helsinki Declaration. Informed consent was obtained from all participants prior to their inclusion in the study. Importantly, this research did not receive any external funding.

Data analysis

Data collected through the questionnaire were exported to Microsoft Excel for initial processing, then analyzed using SPSS software version 25.0.0 (IBM Corp., Armonk, NY). The Shapiro-Wilk test assessed data distribution normality. Descriptive statistics summarize normally distributed variables as means ± standard deviations and categorical variables as percentages and absolute numbers. Independent-sample t-tests or Mann-Whitney U tests, along with chi-square or Fisher's exact tests, were employed to explore differences between variables. Logistic regression analysis was used to identify significant predictors of adverse skin reactions, with significance set at p<0.05. No post-hoc analysis was done. BMI cut-off was used as per Southeast Asian criteria.

## Results

A Google Forms questionnaire was given to 300 participants: 100 faculty, 100 residents, and 100 NOs, aiming to analyze adverse skin reactions due to PPE among HCWs. Overall, 240 participants (80%) responded. The three groups had response rates of 80% (faculty), 65% (residents), and 95% (NOs on COVID-19 duty). Importantly, no data points were missing in the collected responses.

**Figure 1:**
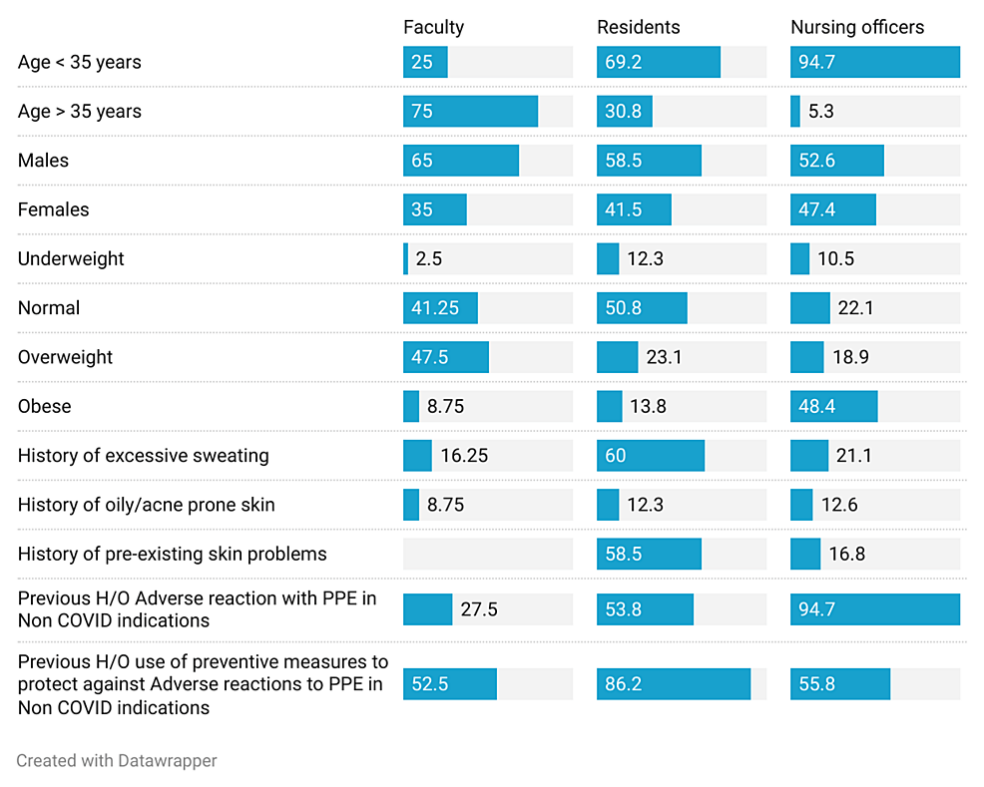
Distribution of the study participants on the basis of baseline demographics H/O=History of, PPE=Personal Protective Equipment

Participants had an average age of 35 years (25 to 50 range), showing significant age differences across faculty, residents, and NOs. Most faculty members were above 35, while residents and NOs were mainly below 35. Males were more common in all categories. Among faculty, 47.5% were overweight, and 48.4% of NOs were obese, with varied BMI. Excessive sweating affected 60% of residents, 21.1% of NOs, and 16.25% of faculty. Around 12% of residents and NOs had oily or acne-prone skin history. Residents (58.5%) had high pre-existing skin issue rates. Preventive measures were common among residents (86.2%). Almost all NOs (95%) experienced PPE-related adverse reactions in non-COVID contexts. Significant differences were found across the three categories (Faculty, Residents, NOs) in terms of age, BMI, excessive sweating history, pre-existing skin problems, prior adverse reactions with PPE, and use of preventive measures (p-values from chi-square tests). Gender and history of oily/acne-prone skin were similar across categories (Table 1, Figure 1).

**Table 1:** Baseline demographics of study populations BMI=Body Mass Index, H/O=History of, PPE=Personal Protective Equipment Chi-square applied to check the associations.

Demographic features, n (%)	Faculty (n= 80) (%)	Residents (n= 65) (%)	Nursing officers (n=95) (%)	p value
Age (Years)				
< 35 years	20 (25)	45 (69.2)	90 (94.7)	<0.0001
> 35 years	60 (75)	20 (30.8)	5 (5.3)	
Gender				
Males	52 (65)	38 (58.5)	50 (52.6)	0.254
Females	28 (35)	27 (41.5)	45 (47.4)	
BMI				
Underweight	2 (2.5)	8 (12.3)	10 (10.5)	<0.0001
Normal	33 (41.25)	33 (50.8)	21 (22.1)	
Overweight	38 (47.5)	15 (23.1)	18 (18.9)	
Obese	7 (8.75)	9 (13.8)	46 (48.4)	
History of excessive sweating				
Yes	13 (16.25)	39 (60)	20 (21.1)	<0.0001
No	67 (83.75)	26 (40)	75 (78.9)	
History of oily/acne prone skin				
Yes	7 (8.75)	8 (12.3)	12 (12.6)	0.685
No	73 (91.25)	57 (87.7)	83 (87.4)	
History of Preexisting skin problem (unrelated to PPE)				
Yes	10 (12.5)	38 (58.5)	16 (16.8)	<0.0001
No	70 (87.5)	27 (41.5)	79 (83.2)	
Previous H/O Adverse reaction with PPE in Non COVID indications				
Yes	22 (27.5)	35 (53.8)	90 (94.7)	<0.0001
No	58 (72.5)	30 (46.2)	5 (5.3)	
Previous H/O use of preventive measures to protect against Adverse reactions to PPE in Non COVID indications				
Yes	42 (52.5)	56 (86.2)	53 (55.8)	<0.0001
No	38 (47.5)	9 (13.8)	42 (44.2)	

Significant differences (p<0.05) in PPE usage during duty hours existed among the three groups, along with associations between the average duration of PPE wear and consecutive days of usage (p<0.05). Over 90% of faculty and NOs wore PPE for over six hours daily. Most faculty members (65%) wore PPE continuously (due to workload) for an average of two days, while NOs averaged 3 days. N95 masks were the most common PPE worn after duty hours for all groups. Goggles, gowns, gloves, and shoe covers were worn by all faculty, residents, and NOs. Caps were used by 100% of NOs, while N95 masks were used by 90.5% of NOs, 71.25% of faculty, and 66.2% of residents (Table 2).

**Table 2:** Type, duration, and frequency of wearing PPE PPE=Personal Protective Equipment Chi-square test was used to find out the association

PPE Variable	Faculty (n= 80) (%)	Residents (n= 65) (%)	Nursing officers (n=95) (%)	P-value
Type of PPE (During duty hours), n (%)				
Respirator mask	24 (30)	27 (41.5)	9 (9.5)	<0.0001
N95 mask	57 (71.25)	43 (66.2)	86 (90.5)	
Cap	70 (87.5)	55 (84.6)	95 (100)	
Goggles	80 (100)	65 (100)	95 (100)	
Face Shield	59 (73.75)	45 (69.2)	81 (85.3)	
Gloves (Double layer)	80 (100)	65 (100)	95 (100)	
Gowns	80 (100)	65 (100)	95 (100)	
Shoe cover	80 (100)	65 (100)	95 (100)	
Average wearing time in hours/day, n (%)				
<6 hours / day	6 (7.5)	22 (33.8)	8 (8.4)	<0.0001
>6 hours / day	74 (92.5)	43 (66.2)	87 (91.6)	
Number of days of continuous use of PPE, on an average, n (%)				
2 days	52 (65)	8 (12.3)	10 (10.5)	<0.0001
3 days	15 (18.75)	29 (44.6)	53 (55.8)	
4 days	13 (16.25)	28 (43.1)	32 (33.7)	
Most common PPE worn beyond duty hours on an average, n (%)				
Respirator mask	6 (7.5)	15 (23.1)	14 (14.7)	<0.0001
N95 mask	76 (95)	58 (89.2)	91 (95.8)	
Cap	28 (35)	22 (33.8)	15 (15.8)	
Gloves	6 (7.5)	37 (56.9)	12 (12.6)	

**Figure 2:**
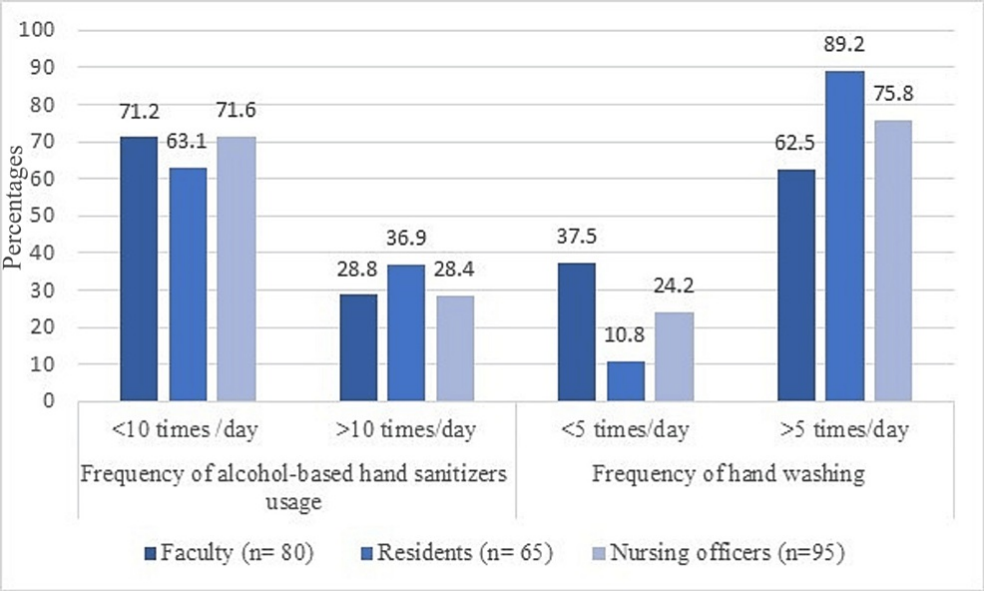
Practice of using sanitizers and hand wash among faculty, residents, and nursing officers Y-axis=Percentages of the participants

The majority of the participants reported using alcohol-based hand sanitizers less than 10 times per day, and this difference was not found to be significant. However, there was a significant difference in the frequency of hand washing among the three groups (p<0.05). Approximately 89% of residents, 75% of NOs, and 62% of faculty members reported washing their hands more than five times per day (Table 3, Figure 2).

**Table 3:** Practice of using sanitizers and hand wash Chi-square test was used to find out the association.

Frequency Variable	Faculty (n= 80) (%)	Residents (n= 65) (%)	Nursing officers (n=95) (%)	P-value
Frequency of alcohol-based hand sanitizers usage				
<10 times /day	57 (71.2)	41 (63.1)	68 (71.6)	0.461
>10 times/day	23 (28.8)	24 (36.9)	27 (28.4)	
Frequency of hand washing				
<5 times/day	30 (37.5)	7 (10.8)	23 (24.2)	<0.001
>5 times/day	50 (62.5)	58 (89.2)	72 (75.8)	

Most participants occasionally used preventive measures to counter PPE-related skin reactions, with similar practices among HCW groups. Proper fitting PPE and avoiding tight clothing were common practices for all. Removing accessories before PPE donning was done by over 80%. Around 90% of NOs bathed frequently, while 60% of all groups adapted mask usage methods to prevent pressure issues. Hand cream use was limited, but about 35% of residents used it before gloves. Around 26% of residents used gels as a skin-PPE interface. Strategies for initial skin indentations included alternative masks or gauze layers (26.2% residents), moisturizing and dressing (19% NOs), and hydropathic compress (13.85% residents, 11.65% NOs, 10% faculty) (Table 4).

**Table 4:** Measures employed to prevent adverse dermatological reactions with PPE PPE=Personal Protective Equipment Chi-square test was used to find out the associations.

Preventive measures against dermatological reactions	Faculty (n= 80) (%)	Residents (n= 65) (%)	Nursing officers (n=95) (%)	P-value
Routine use of preventive measures				
Never	11 (13.7)	8 (12.3)	6 (6.3)	0.475
Occasionally	62 (77.5)	52 (80)	83 (87.4)	
Regularly	7 (8.8)	5 (7.7)	6 (6.3)	
Specific preventive measures employed with regular cream vs Vitamin E plus other emollients				
Use of hand cream before donning gloves as an Interface between PPE and skin	14 (17.5)	23 (35.4)	15 (15.8)	0.521
Use of hand emollients containing hyaluronic acid, ceramide or vitamin E before donning gloves	5 (6.3)	6 (9.2)	8 (8.4)	
Removal of all jewelry, watches and other accessories before donning PPE to prevent pressure ulcers	68 (85)	54 (83.1)	89 (93.7)	0.0801
Frequent bathing	37 (46.3)	42 (64.6)	86 (90.5)	<0.001
Good quality PPE	80 (100)	65 (100)	95 (100)	NA
Properly fitting PPE/goggles	80 (100)	65 (100)	95 (100)	NA
Avoiding tight clothing	80 (100)	65 (100)	95 (100)	NA
Use of masks in alternate ways to prevent pressure induced changes	55 (68.8)	45 (69.2)	75 (78.9)	0.235
Application of gel/moisturizer /appropriate dressing before wearing facial protective equipment as an Interface between PPE and skin	14 (17.5)	17 (26.2)	9 (9.5)	0.020
Management of initial skin indentation				
Management of initial skin indentation with appropriate moisturizing and dressing.	6 (7.5)	5 (7.7)	18 (18.9)	0.212
Management of initial skin indentation with hydropathic compress.	8 (10)	9 (13.8)	11 (11.6)	
Use of other masks or materials or two layers of gauze inside the mask once a single itch or stinging appears	14 (17.5)	17 (26.2)	14 (14.7)	0 .181
Application of moisturizers containing oil control ingredients before and after using of masks to prevent aggravation of existing acne	5 (6.3)	3 (4.6)	7 (7.4)	0.779
Skin cleansing hydration, and moisturisation after removal of PPE	6 (7.5)	3 (4.6)	6 (6.3)	0.775

**Figure 3:**
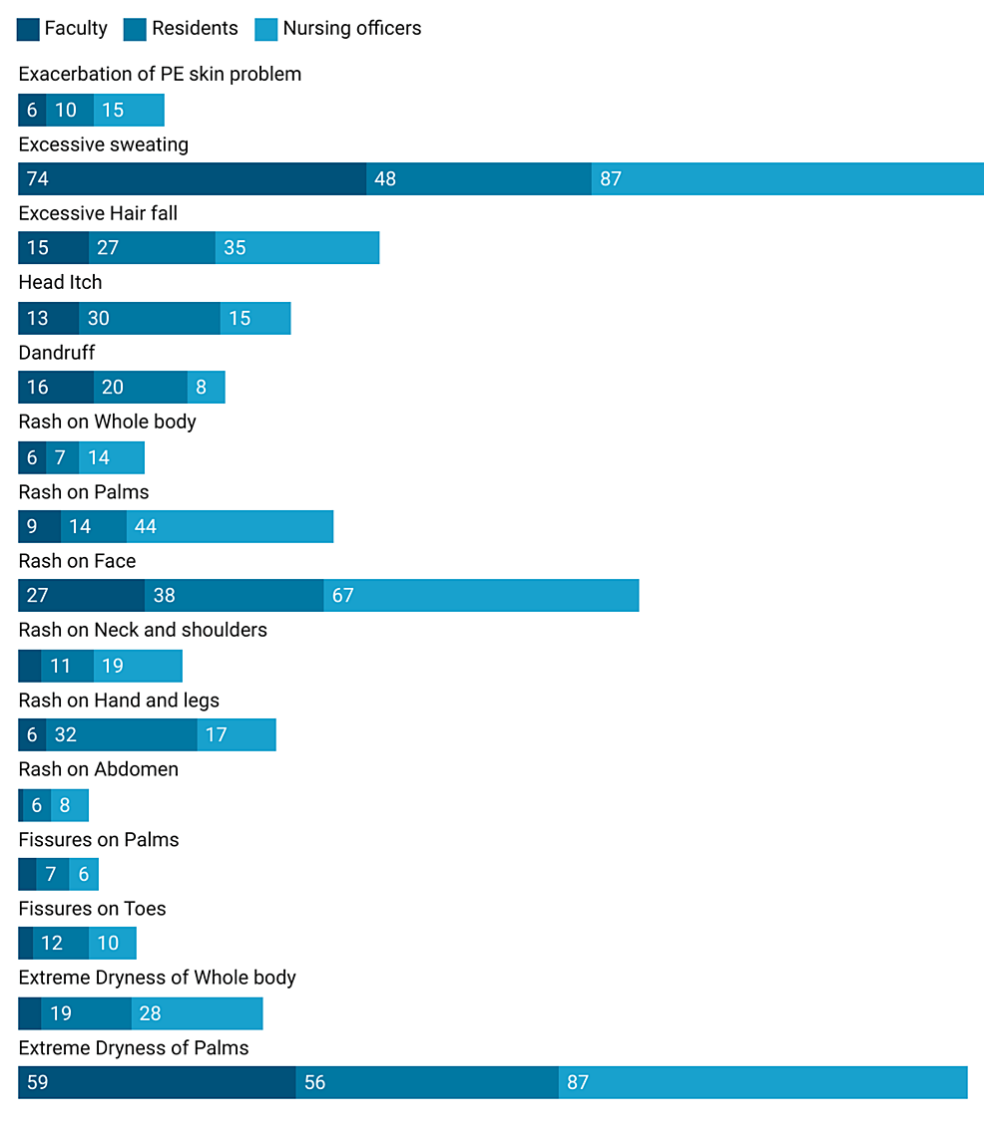
General adverse reactions after PPE usage PE=Pre-Existing, PPE=Personal Protective Equipment

Excessive sweating was the most common dermatological adverse reaction after PPE usage. The development of rash was more common on the face followed by palms. Development of extreme dryness over the palms was another commonly reported ADR, reported by more than 70% HCPs. Over 70% of NOs, 58% of residents, and 30% of faculty complained about rashes on the face after wearing PP (Table 5, Figure 3).

**Table 5:** General adverse dermatological reactions reported after PPE usage PPE=Personal Protective Equipment

Symptoms, n (%)	Faculty (n= 80) (%)	Residents (n= 65) (%)	Nursing officers (n=95) (%)
Exacerbation of PE skin problem	6 (7.5)	10 (15.4)	15 (15.8)
Excessive sweating	74 (92.5)	48 (73.8)	87 (91.6)
Excessive Hair fall	15 (18.8)	27 (41.5)	35 (36.8)
Head Itch	13 (16.3)	30 (46.2)	15 (15.8)
Dandruff	16 (20)	20 (30.8)	8 (8.4)
Development of Rash			
Whole body	6 (7.5)	7 (10.8)	14 (14.7)
Palms	9 (11.3)	14 (21.5)	44 (46.3)
Face	27 (33.8)	38 (58.5)	67 (70.5)
Neck and shoulders	5 (6.3)	11 (16.9)	19 (20)
Hand and legs	6 (7.5)	32 (49.2)	17 (17.9)
Abdomen	1 (1.3)	6 (9.2)	8 (8.4)
Fissure			
Palms	4 (5)	7 (10.8)	6 (6.3)
Toes	3 (3.8)	12 (18.5)	10 (10.5)
Development of Extreme Dryness			
Whole body	5 (6.3)	19 (29.2)	28 (29.5)
Palms	59 (73.8)	56 (86.2)	87 (91.6)

**Figure 4:**
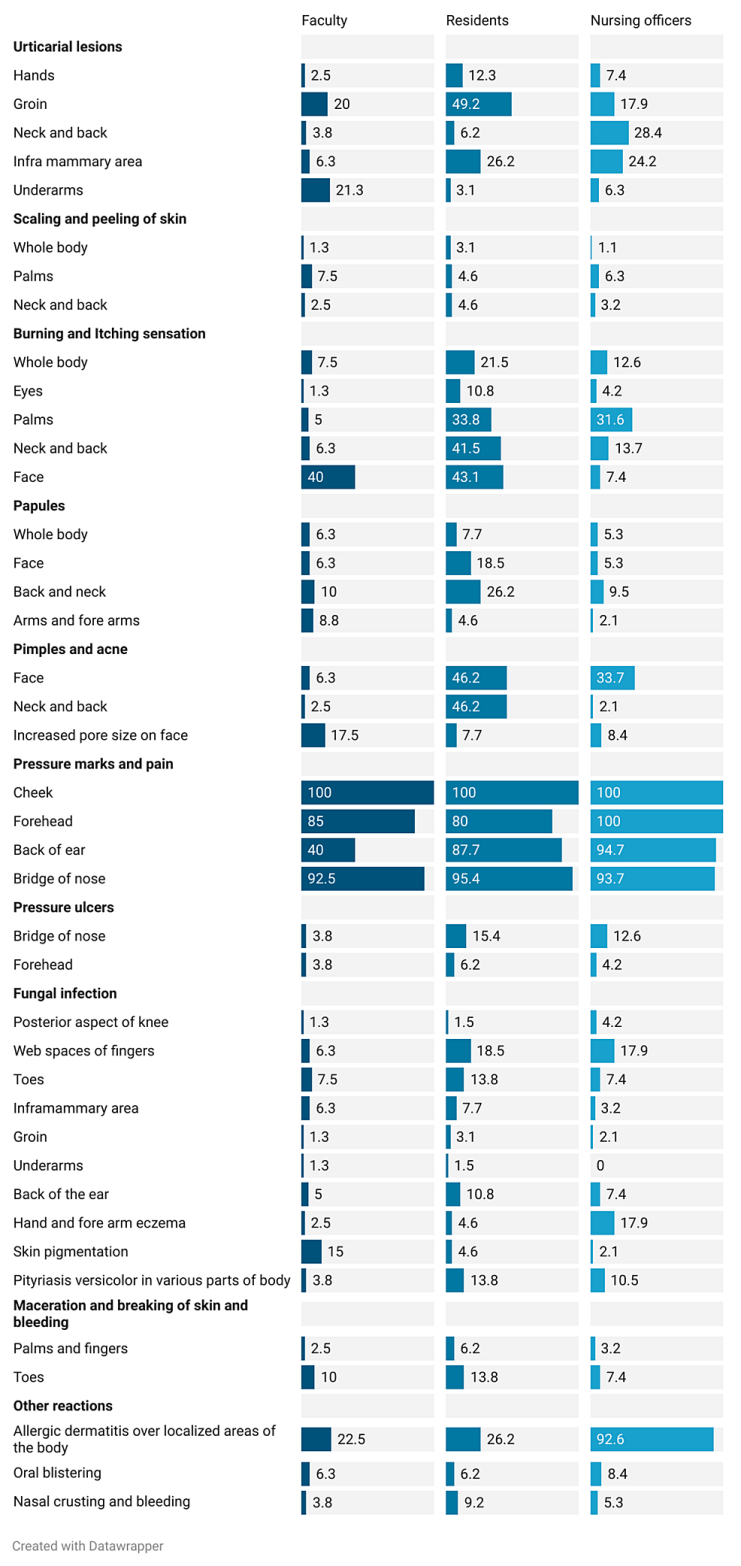
Special adverse dermatological reactions reported after PPE usage among faculty, residents, and nursing officers PPE=Personal Protective Equipment

Around 50% of residents had urticarial lesions near the groin, and 25% of residents and NOs experienced similar lesions in the inframammary area. Over 40% of residents felt burning and itching on their face, neck, and back. About 40% of faculty and 31% of NOs reported similar sensations on their face and palms. Back and neck papules were seen in 26.2% of residents. Scaling, peeling, and acne affected some participants. Pressure marks and pain in the cheeks were reported by all HCWs, with NOs also mentioning it on foreheads. Over 90% suffered nose bridge pressure marks, and 15.4% of residents had nose bridge pressure ulcers. Fungal infections in finger webs were reported by 18% of residents and NOs. Almost 14% of residents had maceration and skin bleeding on their toes. Over 90% of NOs had allergic dermatitis. Occasional oral blistering and nasal crusting were noted. Differences existed across HCP categories for urticarial lesions, sensations, acne, pressure marks, fungal infections, and skin issues (Table 6, Figure 4).

**Table 6:** Special adverse dermatological reactions reported after PPE usage

Symptoms, n (%)	Faculty (n= 80) (%)	Residents (n= 65) (%)		Nursing officers (n=95) (%)		P-value
Urticarial lesions						
Hands	2 (2.5)		8 (12.3)		7 (7.4)	<0.0001
Groin	16 (20)		32 (49.2)		17 (17.9)	
Neck and back	3 (3.8)		4 (6.2)		27 (28.4)	
Infra mammary area	5 (6.3)		17 (26.2)		23 (24.2)	
Underarms	17 (21.3)		2 (3.1)		6 (6.3)	
Scaling and peeling of skin						
Whole body	1 (1.3)		2 (3.1)		1 (1.1)	0.765
Palms	6 (7.5)		3 (4.6)		6 (6.3)	
Neck and back	2 (2.5)		3 (4.6)		3 (3.2)	
Burning and Itching sensation						
Whole body	6 (7.5)		14 (21.5)		12 (12.6)	<0.0001
Eyes	1 (1.3)		7 (10.8)		4 (4.2)	
Palms	4 (5)		22 (33.8)		30 (31.6)	
Neck and back	5 (6.3)		27 (41.5)		13 (13.7)	
Face	32 (40)		28 (43.1)		7 (7.4)	
Papules						
Whole body	5 (6.3)		5 (7.7)		5 (5.3)	0.312
Face	5 (6.3)		12 (18.5)		5 (5.3)	
Back and neck	8 (10)		17 (26.2)		9 (9.5)	
Arms and fore arms	7 (8.8)		3 (4.6)		2 (2.1)	
Pimples and acne						
Face	5 (6.3)		30 (46.2)		32 (33.7)	<0.0001
Neck and back	2 (2.5)		30 (46.2)		2 (2.1)	
Increased pore size on face	14 (17.5)		5 (7.7)		8 (8.4)	
Pressure marks and pain						
Cheek	80 (100)		65 (100)		95 (100)	0.0125
Forehead	68 (85)		52 (80)		95 (100)	
Back of ear	32 (40)		57 (87.7)		90 (94.7)	
Bridge of nose	74 (92.5)		62 (95.4)		89 (93.7)	
Pressure ulcers						
Bridge of nose	3 (3.8)		10 (15.4)		12 (12.6)	0.512
Forehead	3 (3.8)		4 (6.2)		4 (4.2)	
Fungal infection						
Posterior aspect of knee	1 (1.3)		1 (1.5)		4 (4.2)	0.0002
Web spaces of fingers	5 (6.3)		12 (18.5)		17 (17.9)	
Toes	6 (7.5)		9 (13.8)		7 (7.4)	
Inframammary area	5 (6.3)		5 (7.7)		3 (3.2)	
Groin	1 (1.3)		2 (3.1)		2 (2.1)	
Underarms	1 (1.3)		1 (1.5)		0 (0)	
Back of the ear	4 (5)		7 (10.8)		7 (7.4)	
Hand and fore arm eczema	2 (2.5)		3 (4.6)		17 (17.9)	
Skin pigmentation	12 (15)		3 (4.6)		2 (2.1)	
Pityriasis versicolor in various parts of body	3 (3.8)		9 (13.8)		10 (10.5)	
Maceration and breaking of skin and bleeding						
Palms and fingers	2 (2.5)		4 (6.2)		3 (3.2)	<0.001
Toes	8 (10)		9 (13.8)		7 (7.4)	
Other reactions						
Allergic dermatitis over localized areas of the body	18 (22.5)		17 (26.2)		88 (92.6)	0.928
Oral blistering	5 (6.3)		4 (6.2)		8 (8.4)	0.807
Nasal crusting and bleeding	3 (3.8)		6 (9.2)		5 (5.3)	0.358

Among the various factors contributing to adverse skin reactions, pre-existing skin problems and having oily/acne-prone skin were statistically significant (p<0.05), while other factors did not show significant associations. Approximately 33% of individuals with pre-existing skin problems developed adverse skin reactions, whereas only around 6% of those without pre-existing skin problems experienced such reactions (Table 7).

**Table 7:** Association of various factors with adverse skin reaction

Factors		Adverse skin reaction occurred (%)	No Adverse skin reaction (%)	Pvalue
Age	<35 years	74 (47.7)	81(52.3)	0.922
	>35 years	31(36.5)	54 (63.5)	
Gender	Male	22 (15.7)	118 (84.3)	0.013
	Female	29 (29)	71 (71)	
BMI	Normal	11 (12.6)	76 (87.4)	0.084
	Overweight/Obese	29 (21.8)	104 (78.2)	
Pre-existing skin problem	Yes	21 (32.8)	43 (67.2)	<0.0001
	No	11 (6.3)	165 (93.7)	
Previous H/O ADR during non-COVID time	Yes	31 (21.1)	116 (78.9)	0.658
	No	11 (11.8)	82 (88.2)	
Oily/Acne prone skin	Yes	9 (33.3)	18 (66.7)	<0.0001
	No	22 (10.3)	191 (89.7)	

## Discussion

Adverse reactions linked to PPE use are common during COVID-19 and beyond, exacerbated by over-tightening and extended wear. Healthcare organizations must prioritize addressing these issues through proper training and support [4,11]. To explore these concerns, our study analyzed adverse reactions to PPE in different categories of HCWs and their awareness of preventive measures.

In our study, key variables significantly affecting reaction occurrence varied across HCP categories: faculty, residents, and NOs. These variables encompassed age, BMI, excessive sweating history, pre-existing skin problems, prior adverse reactions to PPE, and preventive measures history. Conversely, gender and oily/acne-prone skin history showed no significant inter-category differences. The participants' average age was 35 (range: 25-50) years, displaying notable age discrepancies within the faculty, residents, and NOs groups. Faculty members skewed above 35, while residents and NOs skewed below. Males outnumbered females across all categories. Research has highlighted age and gender's impact on adverse PPE reaction likelihood. A study in the Civil Hospital, Ahmedabad, discovered a higher prevalence of PPE-related adverse effects among 20-40-year-old HCWs [12]. Furthermore, female HCWs exhibited more PPE-related reactions than males. These findings suggest younger age and female gender as potential vulnerability factors linked to differing skin physiology and hormones. Thus, educational training for prevention and treatment should target these groups.

Around 12% of residents and NOs reported prior oily/acne-prone skin in our study. Residents had a high rate (58.5%) of unrelated pre-existing skin problems. HCWs with prior skin issues, oily/acne-prone skin, and an excessive sweating history face an elevated risk of PPE-related reactions. PPE usage can worsen existing skin problems, causing discomfort and complications. Thus, HCWs with prior skin issues need focused care and measures to counter PPE's skin impact [13].

In this study, most faculty members (47.5%) were overweight, and 48.4% of non-physician staff were obese, with significant BMI differences across the groups. Conditions like obesity, diabetes, and smoking elevate the risk of adverse PPE-related events in HCWs. Incorporating HCWs' health status into PPE protocols and offering tailored guidance and training can reduce PPE-related reactions [14].

Over 90% of our study's participants wore PPE for 6+ hours daily, often using N95 masks after hours. Among faculty, 65% wore PPE for about two days continuously, while most non-physician staff used it for approximately three days. Adverse reactions in HCWs are tied to PPE type, duration, and frequency. Studies link skin issues to PPE use over four hours daily and three days weekly, exacerbated by a lack of recovery time and proper skincare. Implementing breaks and PPE rotation reduces reactions. PPE selection is important; tight N95 masks irritate, and non-breathable gowns lead to heat-related problems. Frequent donning, doffing, and extended glove use harm the skin. PPE choice and usage duration should suit tasks and comfort [15].

Most participants used hand sanitizer under 10 times daily, showing no significant difference. Handwashing frequency varied notably among groups (p<0.05). Adverse skin reactions from PPE and hand hygiene products are common among HCWs during the pandemic, resulting in dermatitis, dryness, and itching. A systematic review revealed mask-related cutaneous events at 57.71% and glove/hand hygiene events at 49.16%, including contact dermatitis, acne, and itching [16]. Frequent sanitizer use can lead to redness, dryness, and peeling. Handwashing is recommended for heavy soil or when sanitizers may be ineffective. Proper hygiene and hand care are vital to minimize reactions and protect HCWs and the public during the pandemic [17].

To counter PPE-related HCW issues, preventive measures prioritize well-being and infection control. This study highlighted the intermittent use of strategies against PPE-related skin reactions across healthcare categories. Optimal practices include well-fitting PPE, pre/post-PPE facial cleansing, lightweight non-comedogenic moisturizers, avoiding pore-clogging products, makeup reduction, breaks, washing reusable PPE, barrier products (silicone gel, hydrocolloid), and professional advice. A comprehensive approach involving proper PPE, training, and mental health support effectively mitigates the effects. Skin care includes hydrogel, hydrocolloid, and foam dressings for pressure reduction, along with regular cleansing, moisturizing, soluble moisturizers, and squalane oils to maintain skin barrier health in closed equipment environments [18-29].

Our study extensively characterized common adverse effects in HCPs. Excessive sweating, facial rash, and dry palms were the primary issues after PPE use. Among HCPs, including NOs (70%), residents (58%), and faculty (30%), facial rashes affected around 70%. Urticarial lesions appeared near the groin (50%) and inframammary area (25%). Itching affected 40% of residents on the face, neck, and back. Papules occurred in 26.2% of residents' necks and backs, while 45% showed acne on the face, back, and neck. Pressure marks and pain were prevalent, particularly on NOs' cheeks and foreheads. Nose bridge issues affected over 90%. Fungal infections, skin breakdown, and bleeding varied among HCP categories. Recent literature highlights multiple adverse reactions in HCWs from prolonged PPE use during the pandemic, primarily on the skin and respiratory system. Studies cite pressure-related lesions, contact dermatitis, xerosis, and erythema, especially on the face and hands due to extended mask and glove use. Prolonged PPE application can over-tighten masks, causing tissue damage and irritation. Discomfort and pain from mask straps, as well as respiratory symptoms like breathing difficulties and headaches, have been reported. These reactions impact HCWs' well-being and work performance [4,6,12].

Pre-existing skin problems and oily/acne-prone skin were significant contributors to adverse reactions (p<0.05). At the same time, other factors lacked significant associations in this study, as seen in several other studies [28,29]. A Danish study on HCWs using face PPE during COVID-19 found higher adverse skin reaction rates in HCWs with existing skin conditions. ASR prevalence varied by PPE type; surgical masks caused spots, while FFP3 masks led to redness. This highlights the connection between pre-existing skin issues and PPE-induced reactions.

Limitations

While this study provides valuable insights into the adverse skin reactions associated with PPE usage among HCWs during the COVID-19 pandemic, several limitations warrant mention and consideration. Firstly, the study's cross-sectional design limits our ability to establish causality between PPE usage and adverse skin reactions. Longitudinal studies would be required to observe the progression of skin reactions over time and establish a causal link. Secondly, the sample size and setting of the study, conducted in a single tertiary care COVID-19 hospital, may limit the generalizability of the findings. HCWs in different settings or regions might experience varied types and frequencies of PPE-related skin reactions due to differences in PPE protocols, climate, and types of PPE used. Expanding the study to include multiple hospitals in diverse geographical locations could enhance the representativeness of the results. The response bias may arise due to different perceptions and practices of HCWs towards PPE usage during a pandemic. The low response rate among residents was due to their heavy workload but it has not impact on results.

## Conclusions

The study's findings emphasize the importance of ongoing research and effective strategies to manage adverse reactions among HCWs using PPE during the pandemic. With identified risk factors including pre-existing skin conditions (32.8%), age (47.7% below 35 years), gender (29% female), and PPE misuse, raising HCW awareness about these challenges is crucial. Educating them about proper PPE use, avoiding excessive layers, and ensuring correct fitting to prevent friction-related problems is essential. Widely accepted practices like regular moisturization, particularly in high-friction areas, can maintain the skin barrier and reduce reactions. Timely identification and monitoring of initial signs of adverse reactions allow prompt intervention, lessening discomfort and complications. Promoting skin health awareness, informed PPE practices, and preventive measures can create a safer environment for frontline HCWs.

## Appendices

The link to the questionnaire for the study.

https://docs.google.com/forms/d/e/1FAIpQLSc4OYA6ERbmwyb7rjy1r4051cY2KWzRpaNlMobjlGnfXA2LGA/viewform
